# Growth of Human Colorectal Cancer SW1116 Cells Is Inhibited by Cytokine-Induced Killer Cells

**DOI:** 10.1155/2011/621414

**Published:** 2010-12-01

**Authors:** Yao Wang, Hanren Dai, Hong Li, Haiyan Lv, Tao Wang, Xiaobing Fu, Weidong Han

**Affiliations:** ^1^Department of Immunology, Institute of Basic Medicine, School of Life Sciences, Chinese PLA General Hospital, Beijing 100853, China; ^2^Department of Health Medical Center, Chinese PLA General Hospital, Beijing 100853, China; ^3^Department of Thoracic Surgery, Chinese PLA General Hospital, Beijing 100853, China

## Abstract

Previous reports have suggested that treatment with cytokine-induced killer (CIK) cells may benefit patients with various types of tumor. The aim of this study was to evaluate the antitumor effects of CIK cells against the colorectal cancer line SW1116 *in vitro* and *in vivo*. CIK cells were generated routinely from peripheral blood mononuclear cells of healthy human donors, and the number of CD3^+^CD56^+^ cells was expanded more than 1300-fold after 14-day culture. At an effector : target cell ratio of 50 : 1, the percentage lysis of SW1116 cells reached 68% in the presence of CIK cells, Experimental mice injected with SW1116 cells subcutaneously were divided randomly into four groups: untreated, 5-fluorouracil (5-FU)-treated, CIK-consecutive treated (injected once/day) and CIK-interval treated (injected once every 5 days). CIK cells were injected abdominally five times in total. Compared with the untreated group, xenograft growth was inhibited greatly by CIK treatment, to nearly the same extent as with 5-FU treatment. We demonstrated that the necrotic area in the tumor xenograft was markedly larger in the CIK-treated groups than in the other groups. These findings suggest that CIK-based immunotherapy may represent an effective choice for patients with colorectal cancer.

## 1. Introduction

Colorectal cancer is the third most common cause of death due to cancer in the Western world [1]. In 2009, it was estimated that 75,590 men and 71,380 women were diagnosed with colorectal cancer in the United States [2]. Despite major advances in medical technology and therapy, colorectal cancer still only has an overall 5-year survival rate of 20%–50%. The disease is characterized by the development of a tumor in the large bowel that then spreads throughout the body. Although the primary tumor can be treated by only surgery, treatment of metastases requires some form of adjuvant therapy, such as radioimmunotherapy or chemotherapy. New therapeutic methods are needed to prolong survival. 

Adoptive cellular immunotherapy involves the transfer of immune cells that have been expanded and activated *ex vivo* into patients to eliminate cancer cells. This approach is becoming an important effective method for cancer therapy. In recent years, the application of cytokine-induced killer (CIK) cells has evolved from experimental observations into early clinical studies. These cells have been shown to have encouraging preliminary efficacy towards susceptible autologous and allogeneic tumor cells in both therapeutic and adjuvant settings. CIK cells have a high rate of proliferation; they are derived from peripheral blood mononuclear cells (PBMCs) and are cultured with interferon-*γ* (INF-*γ*), anti-CD3 antibodies, and interleukin (IL)-2 [3, 4]. Among CIK cells, CD3^+^CD56^+^ cells are the main effector cells and demonstrate the most potent cytolytic activity [3, 5]. They have been described as highly efficient cytotoxic effector cells that are capable of recognizing and lysing tumor cell targets in a nonmajor histocompatibility complex-(MHC-)restricted fashion [6, 7]. CIK cells have been shown to target a variety of types of tumor and can exert their cytotoxic effects following systemic delivery [8].

 CIK cells have been found to be highly effective at purging autologous bone marrow in patients with chronic myelogenous leukemia [9]. The antitumor effect of CIK cells has also been observed on many solid tumors, such as hepatoma, lung, and gastric cancers [10–12]. Furthermore, CIK cells can improve the immune function and clinical symptoms of cancer patients. Importantly, the toxicity of CIK cells is minimal, and there is no graft-versus-host reaction associated with their use [5]. In spite of their beneficial features, the cytotoxic activity of CIK cells against human colorectal cancer cells has not been clearly defined. In the study reported herein, we evaluated the antitumor activity of CIK cells *in vitro *against the human colorectal cancer cell line SW1116 and *in vivo* in a nude mouse xenograft model.

## 2. Materials and Methods

### 2.1. Cell Culture

Human colorectal cancer cells (SW1116) and human glioblastoma cells (U251) were originally obtained from the American Type Culture Collection (ATCC, Rockvile, MD, USA) and cultured in high-glucose Dulbecco's modified Eagle's *medium* (DMEM) supplemented with 10% fetal bovine serum, 100 U/ml penicillin and 100 mg/ml streptomycin in a humidified 5% CO_2_ incubator at 37°C.

### 2.2. Generation of CIK Cells

After the healthy blood donor had given informed consent, 10 ml of blood was collected from each in evacuated tubes that contained heparin. Human PBMCs were isolated from fresh blood by Ficoll-Hypaque density gradient centrifugation. The PBMCs were washed three times, adjusted to a final concentration of 2 × 10^6^ cells/ml with CIK medium (Takara, Japan) supplemented with 0.6% autogeneic serum, and then cultured in 75 cm^2^ culture flasks that had been coated with 8 ml of PBS that contained 5 *μ*g/ml antihuman CD3 monoclonal antibody (Takara, Japan) at 4°C overnight. On day 0 of culture, we added 1000 U/ml recombinant human IFN-*γ* (PeproTech, USA) and 1000 U/ml recombinant human IL-2 (rhIL-2, PeproTech, USA) to the culture medium. The cells were cultured in a humidified 5% CO_2 _ incubator at 37°C. The cells were transferred from the coated flasks to fresh flasks after four days. Every three days, fresh CIK medium and 1000 U/ml rhIL-2 were added. After culture for 14 days, approximately 1 × 10^9^ CIK cells were harvested per flask, with a survival rate of >95%.

### 2.3. Phenotypic Analysis of CIK Cells

A total of 5 × 10^5^ CIK cells were harvested and washed twice with PBS. The cells were resuspended in 100 *μ*l of PBS, labeled with 15 *μ*l of antibodies against CD4/8/3 (FITC-conjugated anti-CD4, PE-conjugated anti-CD8, and PerCP-conjugated anti-CD3; BD, USA) and 5 *μ*l of anti-CD56 antibody (APC-conjugated anti-CD56; BD, USA) in the dark for 30 min at 4°C, and then washed twice. Fluorescence-activated cell sorting (FACS) was then performed. The phenotype of PBMCs was analyzed as a control.

### 2.4. MTT Cytotoxicity Test of CIK Cells In Vitro

SW1116 cells were plated in 96-well plates in triplicate at a density of 4 × 10^4^ cells/well. After the tumor cells had adhered completely, CIK cells were added at different ratios of effector : target (1 : 1, 1 : 5, 1 : 10, 1 : 20, and 1 : 50), in 200 *μ*l of medium without serum. After incubation for 24 hours, the supernatant in each well was removed and the cells washed three times. Aliquots of 100 *μ*l of medium without serum and 10 *μ*l of MTT were mixed and added to each well. After incubation at 37°C for 4 h, the supernatant was removed carefully, and 150 *μ*l of DMSO were added to each well. The cells were then shocked for 10 min in the dark. The OD was assessed by spectrophotometry at a wavelength of 492 nm. As a control, PBMCs were subjected to the same procedure. The amount of cell death was calculated according to the following equation: death rate = (OD_control_ − OD_sample_)/OD_control_ × 100%.

### 2.5. Pathological Observation

SW1116 cells were placed on a slide putted in the culture capsule. When the cells reached 80% confluence, CIK cells were added at an effector :  target ratio of 1 : 20. After incubation for 24 h, the slides were washed twice, stained with hematoxylin and eosin (HE), and sealed with neutral gum. We observed the shapes and aggregation of the cells by light microscopy. Tumor specimens were fixed with 10% neutral formaldehyde solution for 24 h, dehydrated in an ethanol gradient, made transparent with dimethylbenzene, embedded in paraffin, sectioned at a thickness of 3-4 *μ*m, and stained with HE.

### 2.6. Nude Mouse Xenograft Assay

Nude mice were obtained from the Chinese People's Liberation Army Academy of Military Medical Science. The biologic license number was SCXK-(Jun)2007-004. Nude mice were bred in an animal institute that complied with good laboratory practice (Chinese PLA General Hospital Animal Experiment Centre). On day 0, 5 × 10^6^ SW1116 cells were injected subcutaneously into the nude mice. The nude mice were found to have developed 0.2 cm^3^ tumor nodules after 5 days. They were then randomly divided into four groups: the untreated group, 5-fluorouracil-(FU-)treated group, CIK-consecutive treated group, and CIK-interval-treated group. In the 5-FU-treated group, 5-FU was injected intravenously at 50 mg/kg every day for 5 days in total. In the CIK-consecutive treated group, the mice were injected abdominally with CIK cells (5 × 10^7^ cell/day) for 5 days. In the CIK-interval-treated group, 5 × 10^7^ of CIK cells were injected abdominally into mice once every 5 days, that is, 5 times in 3 weeks. Tumor volumes and body weights were measured every 2 days. Tumor volumes were calculated by using the formula: length (mm) × width (mm) × height (mm). On day 30, the mice were sacrificed, and the tumors were weighed. To detect toxicity to the animals, the body weights of the animals were measured. After fixing in formalin, the tumor tissues were stained with HE. Pictures were taken randomly in 10 fields of vision, and image processing software (Image-Pro Plus Version 4.5, USA) was used to calculate the necrotic area.

### 2.7. Statistical Analysis

The results are shown as the mean ± standard error of the mean (SEM) of triplicate determinants (wells). Data were plotted using GraphPad Prism version 5.00. Two-way analysis of variance (ANOVA) was used to determine the significance of the difference between the means of all experiments. A *P* value of less than.05 was considered to be statistically significant.

## 3. Results

### 3.1. Phenotype of the CIK Cells

**Figure 1:**
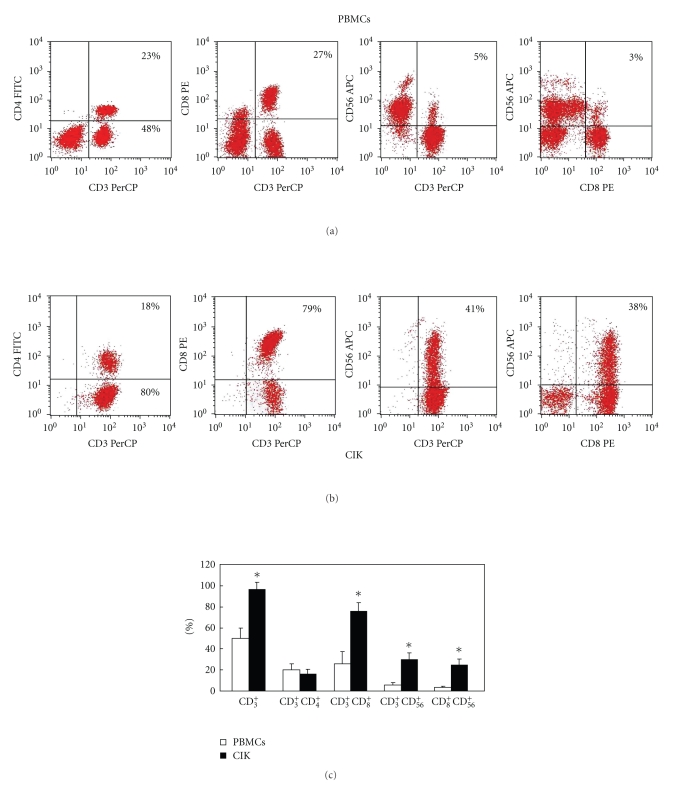
Phenotypic analysis of CIK cells. All cell samples were stained with PerCP-conjugated antibodies against CD3, FITC-conjugated antibodies against CD4, PE-conjugated antibodies against CD8, and APC-conjugated antibodies against CD56. (a) Typical phenotypic analysis of PBMCs. (b) Typical phenotypic analysis of CIK cells. (c) Comparison of the phenotypic analyses of PBMCs and CIK cells. The PBMCs population was composed of 50% CD3^+^ cells, 4% CD3^+^CD56^+^ cells, 27% CD3^+^CD8^+^ cells, 22% CD3^+^CD4^+^ cells, and 3% CD8^+^CD56^+^ cells. After culture for 14 days, the CIK cells comprised 98% CD3^+^ cells, 77% CD3^+^CD8^+^ cells, 20% CD3^+^CD4^+^ cells, 38% CD8^+^CD56^+^ cells, and 41% CD3^+^CD56^+^ cells. Phenotypic comparison analysis was performed from 15 samples, and the results are expressed as means ± SD. (*P < .05)

Firstly, we established a stable system for the expansion of CIK cells *in vitro*. PBMCs from 15 individuals were cultured to generate CIK cells. The phenotypes of the PBMC and CIK cells were examined by FACS. The PBMC population contained 50% CD3^+^ cells, 4% CD3^+^CD56^+^ cells, 27% CD3^+^CD8^+^ cells, 22% CD3^+^CD4^+^ cells, and 3% CD8^+^CD56^+^ cells (Figure 1(a)). After culture for 14 days, the CIK cell population contained 98% CD3^+^ cells, 41% CD3^+^CD56^+^ cells, 77% CD3^+^CD8^+^ cells, and 20% CD3^+^CD4^+^ cells (Figure 1(b)). After 14 days, the counts of the total number of cells was increased by 130-fold. The number of CD3^+^CD56^+^ cells was increased by 1300-fold, whereas the number of killer T cells (CD3^+^CD8^+^) was increased by 390-fold. The counts of the two types of cells were evidently different between the PMBC and CIK cells (Figure 1(c)). The proportion of CD3^+^CD56^+^cells was <5% before culture, but 35% after culture. CD3^+^CD56^+^ cells are the main effector cells; therefore the harvested suspension cells were mature CIK cells. The phenotypes of the CIK cells from the 15 individuals were not evidently different, which demonstrates that the method tested for the culture of CIK cells is reproducible.

### 3.2. Cytotoxicity of CIK Cells In Vitro

**Figure 2:**
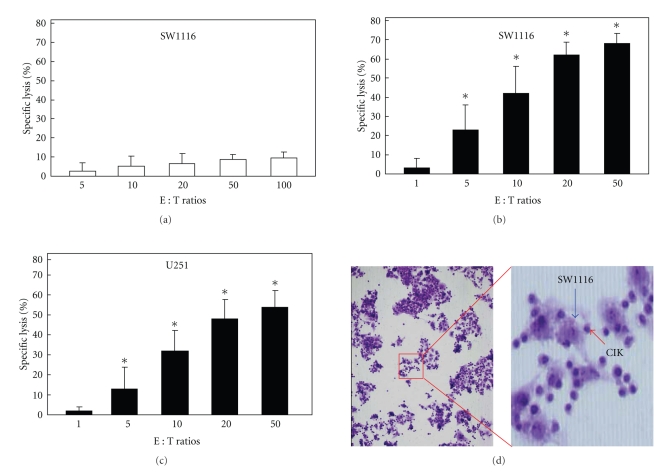
Cytotoxicity of CIK cells* in vitro.* (a) Cytotoxicity of PBMCs against SW1116 cells. (b) Cytotoxicity of CIK cells against SW1116 cells. (c) Cytotoxicity of CIK cells against U251 cells. At an effector : target ratio of 100 : 1, the percentage lysis of SW1116 cells after the addition of PBMCs was 9%. At effector : target ratios of 1 : 1, 5 : 1, 10 : 1, 20 : 1, and 50 : 1, the percentage lysis after the addition of CIK cells was 3%, 23%, 42%, 62%, and 68%, respectively, for SW1116 cells and 2%, 13%, 32%, 48%, and 54%, respectively, for U251 cells. (d) Observation of the distribution of CIK cells by HE staining, ×100 and ×200. CIK cells adsorbed to and aggregated around the SW1116 cells. For cytotoxic assay, each experiment was performed in triplicate and was repeated at least three times, and the results are expressed as mean ± SD. (*P < .05)

Next, we examined the antitumor effect of CIK cells *in vitro*. CIK cells have been shown to demonstrate cytotoxicity in a non-MHC-restricted manner. CIK cells show strong anti-tumor activity against lung cancer, ovarian cancer, cervical cancer, and other types of tumor cells *in vitro* [11–14]. In this study, at an effector:target ratio of 100 : 1, the mean percentage lysis of SW1116 cells was 9% after the addition of fresh PBMCs (Figure 2(a)). At effector : target ratios of 1 : 1, 5 : 1, 10 : 1, 20 : 1, and 50 : 1, the mean percentage lysis after the addition of CIK cells was 3%, 23%, 42%, 62%, and 68%, respectively, for SW1116 cells and 2%, 13%, 32%, 48%, and 54%, respectively, for U251 cells (Figures 2(b) and 2(c)). The CIK cells were suspension cells and therefore could not adsorb to the slide on their own. The cells were observed by HE staining after coculture of the CIK and SW1116 cells for 24 h. The CIK cells were round and had a high proportion of nucleoplasm, whereas the SW1116 cells were irregular and had a low proportion of karyoplasm. The CIK cells adsorbed and aggregated around SW1116 cells (Figure 2(d)). Cytotoxicity tests showed that the CIK cells had a strong ability to kill SW1116 cells as compared with normal lymphocytes. HE staining showed that when CIK cells and tumor cells were cultured together, the CIK cells gathered around the tumor cells without MHC restriction or specificity.

### 3.3. Antitumor Effects of CIK Cells In Vivo

**Figure 3:**
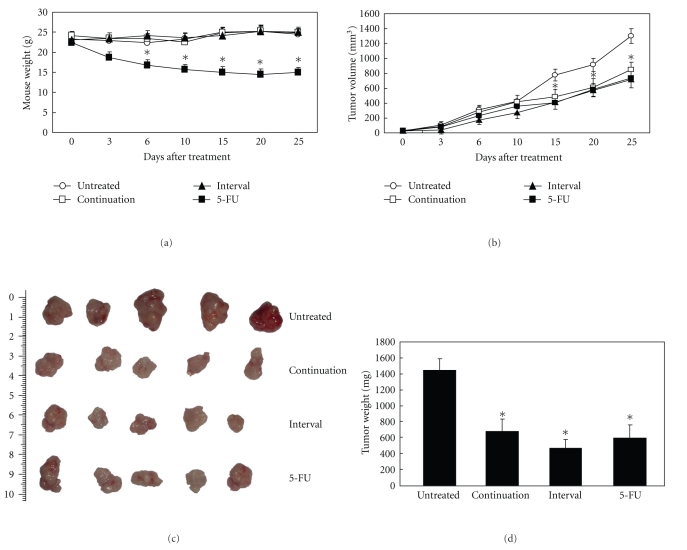
Antitumor effects of CIK cells* in vivo*. Nude mice (*n* = 30) were injected subcutaneously with 5 × 10^6^SW1116 colorectal cancer cells. In the CIK-consecutive-treated group, 5 × 10^8^ CIK cells were injected abdominally into the mice once every day for 5 days. In the CIK-interval-treated group, 5 × 10^8^ CIK cells were injected abdominally into the mice once every 5 days on 5 occasions, namely 5 times in 3 weeks. In the 5-FU-treated group, 5-FU was injected intravenously at 50 mg/kg. Body weights were measured every 2 days (a). Tumor volumes were calculated by using the formula: length (mm) × width (mm) × height (mm) (b). Representative photographs are shown (c). On day 30, the mice were sacrificed, and the tumor weights were determined (d). Standard deviations and *P*-values were calculated with Student's *t*-test (*P* < .05).

Finally, we evaluated the inhibition of growth of colorectal cancer xenografts by CIK cells. The two groups of mice treated with CIK cells showed no signs of panic, irritability, weakness, or other symptoms after CIK cells were injected abdominally. Throughout the treatment period, there was no significant decline in the weight of the mice in these groups, whereas 5-FU showed evident toxicity. After being treated with 5-FU, the some symptoms, for example, moving slowly, urinary and fecal incontinence, were observed in the nude mice. On day 3, the weight of the mice decreased significantly, and two mice died within the treatment period (Figure 3(a)). Preliminary experiments showed that nude mice had significant side effects, in the abdominal cavity after injection of 5-FU, whereas injection of 5 × 10^7^ CIK cells did not result in any toxicity.

**Figure 4:**
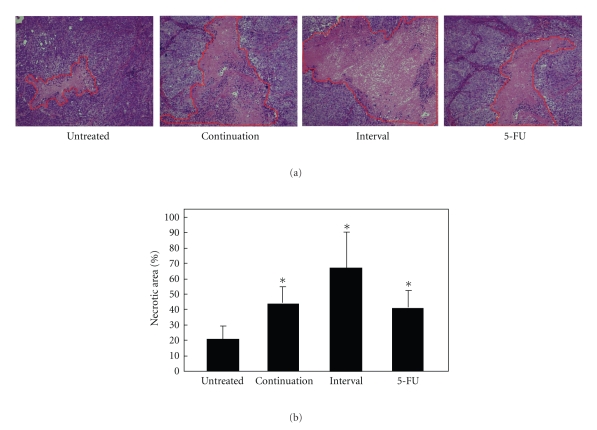
Pathological observation of the necrotic area of the xenografts. HE staining ×200. Representative examples of the necrotic area in the four different groups are shown (a). Necrotic area was calculated with Image-Pro Plus Version 4.5 (b). Standard deviations and *P* values were calculated with Student's *t*-test (*P* < .05).

Through the measurement of tumor volume and tumor weight, we demonstrated a powerful antitumor activity of CIK cells. The consecutive-treated and interval-treated groups showed a reduction in tumor volume of 41% and 52%, respectively, whereas the 5-FU group showed a decrease in tumor volume of 43% (Figure 3(b)). On day 30, the mice were sacrificed and the tumors isolated. In the control group, the mean value of tumor weight increased to 1.448 g at 30 days after injection. In the consecutive-treated and interval-treated groups, tumor growth was inhibited by 53% and 62%, respectively, whereas in the 5-FU-treated group tumor growth was inhibited by 54% (Figures 3(c) and 3(d)). In this experiment, we found that there was a tendency towards better efficacy in the CIK-consecutive-treated group as compared with the CIK-interval-treated group, but there was no significant difference between the two groups. HE staining demonstrated that the necrotic area of the tumor tissues was greater in the groups that had been injected with CIK cells than in the other groups; the necrotic area in the former measured up to 60%, as compared with 24% for the control group (Figures 4(a) and 4(b)). Interestingly, although the CIK cells and 5-FU both significantly inhibited tumor growth, the area of tumor necrosis after treatment with CIK cells was significantly higher than that observed after treatment with 5-FU. Therefore, in the future it will be of interest to investigate the mechanisms by which CIK cells inhibit tumor growth.

## 4. Discussion

Adoptive immunotherapy has now been available for nearly 30 years. One of the first prototypes was the lymphokine-activated killer (LAK) cells. In clinical studies, LAK cells demonstrated modest efficacy against metastatic cancers such as renal cell carcinoma and melanoma [15]. Subsequently, studies confirmed that standard IL-2, stimulated LAK cells had low antitumor activity, and it was difficult to generate large numbers of cells [16–18]. The emergence of LAK cells that accelerated the field of cellular immunotherapy with CIK cells was performed in Stanford [19]. CIK cells possess a higher level of cytotoxic activity and a higher rate of proliferation than LAK cells [20]. Over the past 20 years, the development of CIK cell immunotherapy for the treatment of cancer has received considerable attention. CIK cells exhibit a high rate of proliferation [21]; during the culture period, CD3^+^CD56^+^ cells can expand by up to 1000-fold [3]. In this study, we used culture dishes that had been coated with a monoclonal antibody against CD3. The isolated PBMCs were plated onto the coated dishes, and IFN-*γ* and IL-2 were added to the medium. After four days, the cells were removed from the coated dishes and cultured further in the presence of IL-2. The cells were harvested after 14 days. The total cells expanded by up to 130-fold, and the CD3^+^CD56^+^ cells expanded by up to 1300-fold. The phenotype, composition, and quantity of the cells were as described in other reports [11, 14, 22].

Currently, the mechanisms of the genesis and development of colorectal cancer remain unclear, but it is generally agreed that the action of various factors in the tumor enables the immune system to be evaded and results in unlimited proliferation of tumor cells. Research has shown that in the animal model of colorectal cancer and in patients with colorectal cancer patients, antigen-specific cytotoxic T lymphocytes (CTL) are induced [23]. Therefore, colorectal cancer is immunogenic, but it is possible that colorectal cancer still develops for the following reasons: (1) in patients with colorectal cancer where expression of the histocompatibility leukocyte antigen (HLA)-I was decreased or absent [24], the lack of antigen presentation by the tumor cells led to the induction of CTL responses. (2) Mutations in peptide transporting molecules (TAP) may also affect the presentation of T cell epitopes [25]. (3) Colorectal tumors express not only functional Fas ligand (FasL), which can induce apoptosis in tumor infiltrating T cells that bear Fas, but also Fas itself, which although expressed at lower levels than in normal colon epithelium may make the tumor cells susceptible to apoptosis [26–29]. (4) Surface expression of the CD3 *ζ* (zeta) chain of the T cell receptor (TCR) is decreased in tumor-infiltrating T lymphocytes (TIL) from patients with colorectal cancer. (5) Colorectal cancer cells secrete factors such as transforming growth factor beta (TGF-*β*), which promotes tumor growth, and IL-10, which inhibits the cell immune response. Given that colorectal cancer is caused by the loss of immune function, only a few studies of the treatment of colorectal cancer by immunotherapy have been undertaken. For example, among 30 patients with colorectal cancer treated with LAK cells and IL-2, one complete anti-tumor immune response and four partial anti-tumor immune responses were seen [30]. In addition, 7 patients with metastatic colorectal carcinoma resistant to chemotherapy were treated by transfusing autologous IL-2 modified CIK cells [31]. This pilot study demonstrated an anti-tumor immune response of this approach in at least partial patients. 

Here, we provide evidence that CIK cells might be a good candidate for colorectal cancer therapy. The major effector cells of CIK cells, namely those that show the greatest cytotoxicity, are the subset of CD3^+^CD56^+^ cells [6], which express both the T-cell marker CD3 and natural killer cell marker CD56 and are termed non-MHC-restricted T cells. These cells are capable of killing both autologous and allogeneic tumor targets. The cytotoxicity of CIK cells might be mediated via contact between the adhesion receptor lymphocyte function-associated antigen-l (LFA-l), which is found on all T cells, and its counter receptor, intercellular adhesion molecule-l (ICAM-l), which is located on the surface of the target cells [7]. CIK cells possess cytoplasmic granules which contain the protein perforin (cytolysin). The mechanism of destruction of target cells includes the vectorial exocytosis of the contents of the cytoplasmic granules into the intercellular space by the effector cell at the site of target-cell contact [32, 33]. In the vitro study by HE staining, the result showed CIK cells adhered to the surface of SW1116 cells, and they possessed powerful killing capacity for SW1116 cells. This result was consistent with the killing of tumor cells by CIK cells. CIK cells are effective against FasL-positive malignant cells and cells with multidrug resistance (MDR), and it has been observed that a population of CIK cells migrated to tumor sites by the 7th hour after injection and remained detectable at these sites for an additional 9 days [8, 34]. In agreement with this, we observed *in vivo* that abdominal injection of only 5 × 10^7^ CIK cells resulted in strong inhibition of colorectal tumor growth. Although a similar effect was observed with 5-FU, the CIK cells had low toxicity and few side effects as compared with the chemotherapy drug. In contrast to chemotherapy drugs, which have a suppressive effect on immune cells, CIK cells not only kill tumor cells directly, but also can themselves secrete many cytokines, such as IL-2, TNF-*α*, and granulocyte macrophage colony-stimulating factor (GM-CSF), which enhances the systemic anti-tumor activity of the body. HE staining of tissue biopsies after treatment with CIK cells revealed the presence of a large area of necrosis around the tumor. We speculate that after CIK cells have been injected into the body, they gather first around the tumor, and then a large number of CIK cells infiltrate the tumor and cause necrosis. Further experiments are required to confirm this hypothesis.

## 5. Conclusions

 There are several studies concerning adoptive cellular immunotherapy with many solid tumors, but this is the first study to address colorectal cancer SW1116 cell lines. In this paper, we observed the antitumor activity of CIK cells against human colorectal cancer *in vitro* and *in vivo*. Cells with the phenotype CD3^+^CD56^+^ are rare (1%–5%) in uncultured peripheral blood lymphocytes [6, 19]. The CIK cells possessed the strong proliferation capacity; the CIK cells derived from 50 ml peripheral blood can be amplified three folds as much as the quantity of normal human lymphocytes. The results of the nude mouse xenograft assay showed that the CIK-consecutive-treated and CIK-interval-treated groups experienced a similar inhibitory effect to that observed with 5-FU. These results show that immunotherapy with CIK cells is a suitable adjuvant therapy for colorectal cancer. We propose that different CIK cells treatment programs will be used for different patients in clinical practice.
